# Cilia at the Crossroads of Tumor Treating Fields and Chemotherapy

**DOI:** 10.1159/000529193

**Published:** 2023-01-17

**Authors:** Loic P. Deleyrolle, Matthew R. Sarkisian

**Affiliations:** ^a^Department of Neurosurgery, Adam Michael Rosen Neuro-Oncology Laboratories, University of Florida, Gainesville, Florida, USA; ^b^Preston A. Wells, Jr. Center for Brain Tumor Therapy, University of Florida, Gainesville, Florida, USA; ^c^Department of Neuroscience, McKnight Brain Institute, University of Florida, Gainesville, Florida, USA

**Keywords:** Primary cilia, Temozolomide, TTFields, Glioma, Neural development

## Abstract

Glioblastoma (GBM), the most common and lethal primary brain tumor in adults, requires multi-treatment intervention which unfortunately barely shifts the needle in overall survival. The treatment options after diagnosis and surgical resection (if possible) include irradiation, temozolomide (TMZ) chemotherapy, and now tumor treating fields (TTFields). TTFields are electric fields delivered locoregionally to the head/tumor via a wearable medical device (Optune^®^). Overall, the concomitant treatment of TTFields and TMZ target tumor cells but spare normal cell types in the brain. Here, we examine whether primary cilia, microtubule-based “antennas” found on both normal brain cells and GBM cells, play specific roles in sensitizing tumor cells to treatment. We discuss evidence supporting GBM cilia being exploited by tumor cells to promote their growth and treatment resistance. We review how primary cilia on normal brain and GBM cells are affected by GBM treatments as monotherapy or concomitant modalities. We also focus on latest findings indicating a differential regulation of GBM ciliogenesis by TTFields and TMZ. Future studies await arrival of intracranial TTFields models to determine if GBM cilia carry a prognostic capacity.

## Introduction

The goal of any cancer treatment is to selectively target cancer and spare normal cells. When approaching aggressive brain tumors, like glioblastoma (GBM), most normal, differentiated neural cell types in the brain survive standard-of-care therapy. On most normal neural cell types, one can detect primary cilia (e.g., on neurons or astrocytes) or motile cilia (e.g., on ependymal cells). Primary cilia are microtubule-based but nonmotile “antenna-like” organelles. These organelles not only shape brain development but also potentially protect the brain from various environmental stressors. Primary cilia are also maintained by GBM cells, but the roles of cilia on aggressive brain tumors are only just emerging [1, 2]. After briefly reviewing roles of cilia during normal neural development, we will discuss recent findings that examine how the latest treatments for GBM affect both normal and tumor cilia and how this may in turn affect the organelles' ability to govern growth and treatment resistance of both normal and tumor cells. We focus our attention on tumor treating fields (TTFields) and temozolomide (TMZ) chemotherapy and explore the different ways these treatments affect primary cilia and what this could mean for cell survival in tumor versus normal cell types.

## Normal Neural Cell Types Grow Primary Cilia Which Are Associated with Integration and Cell Survival

After progenitor cells complete mitosis, the mother centriole usually migrates and docks to the plasma membrane and elongates a primary cilium, a structure whose microtubules are in a 9 + 0 arrangement [3]. Ciliary elongation and maintenance are achieved by a process known as intraflagellar transport, which shuttles cargo anterogradely to the cilia tip and retrogradely to the cell body [4]. If a cell divides again, the cilium must be disassembled through mechanisms that are just beginning to be understood [5]. Cilia disassembly is essential because centrioles must be duplicated and segregated for another round of mitosis [6]. While in the ciliated state, cells can translocate specific receptors and signaling mediators to be able to relay signals from the surrounding environment back to the cell body. The formation of cilia and various signaling pathways that localize to nonmotile primary cilia has been reviewed extensively (e.g., [7]). Nevertheless, it is thought these structures and pathways within them are exploited by most, if not all, developing neural cell types. There are several reviews on this topic [8−12], but here we highlight key examples about cilia role in neural growth and survival.

In normal developing brain, primary cilia are believed to host signaling events that shape cell proliferation, migration, and differentiation. In setting up these processes, it appears critical that radial glia (RG) cells, the chief neural stem cells in developing CNS, maintain their cilium along the ventricular surface lining [13]. ARL13B, a regulatory GTPase signaling within RG cilia, appears critical for the polarized cellular scaffold that supports both generation and migration of subsequently neural progenitors. Conditional depletion of *Arl13b* in RG cells early in development reverses the polarity of the RG scaffold, resulting in a developmentally inverted cortex [13]. RG cells give rise to neuronal progenitors that are also transiently ciliated. These cilia are sensitive to sonic hedgehog (SHH) ligand, which can stimulate proliferation of neural stem cells and influence migratory behaviors of neurons as they depart the ventricular zone and head for future gray matter [14−17]. Once migrating neurons reach their destination, they elaborate primary cilia which acquire new functions that regulate outgrowth of neuritic processes and provide signals that ensure integration into normal circuitry [18−23]. For example, most developing neurons enrich type 3 adenylyl cyclase and ARL13B, and conditional deletion of these signaling mediators disrupt dendritic development and synaptic integration into cortex. ARL13B signaling from cilia can shape axon tract development [24], though there is also evidence that ARL13B functions outside primary cilia to regulate axon guidance [25]. Oligodendrocyte precursors also transiently possess a cilium sensitive to SHH, deletion of which can reduce oligodendrogenesis and result in abnormal oligodendrocyte differentiation and impairment of fine motor control [26, 27]. The mechanisms by which cilia control cytoplasmic/transcriptional changes in all of these basic neurodevelopmental steps are not well understood. Other pathways are likely exploited, for example, cilia-dependent pathways that control cAMP or PKA signaling [28] during these developmental steps, but the details of how their signaling works both outside and inside the cilium and downstream of cilia are less clear. However, the data seem clear that disruption of numerous ciliary genes significantly alters all of the above mentioned developmental processes [29−31], implicating conserved functions of cilia to ensure normal neural development.

Interestingly, primary cilia on developing cortical neurons and glia carry potential neuroprotective roles. For example, alcohol and ketamine were shown to stimulate insulin growth factor 1 receptor to cilia and activated downstream AKT signaling [32]. After depleting neuronal cilia postnatally by disrupting *Ift88* expression, neurons in the mouse brain were more susceptible to dendritic degeneration and cell death upon alcohol or ketamine exposure [32]. Similarly, in substantia nigra, it was reported that dopaminergic neuronal cilia mediate mitochondrial and metabolic stress responses [33, 34]. Disrupting ciliogenesis on these neurons facilitates MPTP-induced neuronal loss [33]. In normal astrocytes in vitro, SHH signaling via the primary cilium protected cells from serum-starved, stressed conditions [35]. Therefore, maturing neural cell types throughout the brain exploit signaling pathways regulated within or by their cilium to promote their survival upon variety of environmental challenges.

## Primary Cilia Develop on GBM Cells and Can Mediate Tumorigenesis

Since primary cilia play roles in normal neurogenesis and migration, it would also seem plausible they are exploited by tumor cells during proliferative expansion or invasion around the brain. Some GBM tumors or cell lines lack primary cilia [36, 37]. Loss of primary cilia in some glioma lines has been linked to underlying ultrastructural defects [36, 37] and/or upregulation of negative regulators of ciliogenesis such as BAG3 [38]. Elegant studies have shown that astrocytes concentrate lysophosphatidic acid signaling pathway in their primary cilia, which serves to restrict proliferation and transformation to GBM [39]. If this signaling is re-routed out of the cilium, it can promote astrocyte proliferation, and inhibiting lysophosphatidic acid signaling on de-ciliated astrocytes and GBM cells suppresses their proliferation in vitro and in vivo [39]. In addition, restoring primary cilia to glioma cells that lack cilia triggered loss of self-renewal status and differentiation of the glioma stem cells [40]. Restoring the cilia was also found to reduce their invasive capacity in co-culture experiments with human brain organoids [40].

There is also the possibility that GBM cells exploit their primary cilia as a growth advantage. Primary cilia are found in up to 25–40% of cells in patient biopsies and newly derived lines [40−45]. A recent study showed glioma ciliation is linked to glioma cell stemness and thru the master transcriptional regulator SOX2 and superenhancer KLHDC8A expression [45]. Ablation of KLHDC8A reduced primary cilia, markers of glioma stem cells, and proliferation [45]. The presence of primary cilia is perhaps unsurprising since GBMs are usually of low mutational burden [46, 47] and ciliary mutations are not common compared to other mutations that are frequently observed in GBM (e.g., TP53, PTEN, NF1, IDH1, EGFR) [48]. The primary cilia found on GBM cells are also capable of transduction, mobilizing components of the SHH pathway into/out of the cilium and coordinating downstream target expression in response to SHH ligand [45, 49, 50]. In addition, multiple studies have found that disrupting key ciliary regulators such as KIF3A and ARL13B on GBM cells derived from various subtypes somewhat consistently prolonged survival in tumor-bearing mice [42, 45, 49]. These results suggest GBM cells exploit their cilia to promote tumor growth.

## TTFields Ablate Glioma Primary Cilia but Spare Developing Normal Neural Cell Types

One of the most recent treatment options for patients with GBM is TTFields therapy [51]. Patients wear arrays attached to an electric field generator that they carry around up to 18 h/day during their daily routine. The treatment delivers low-intensity (1–3 V/cm) alternating electric fields across the head/tumor at 200 kHz frequency. When applied concomitantly with standard-of-care TMZ chemotherapy, overall survival is prolonged by 4-5 months [52]. The additional survival time may seem unsignificant, but since the establishment of standard of care (TMZ, gamma irradiation, and surgery) over the past ~20 years [53], it is the greatest extension of survival to date. Understanding how TTFields promote changes to tumors cells is of interest to try and improve the treatment's effects.

**Fig. 1:**
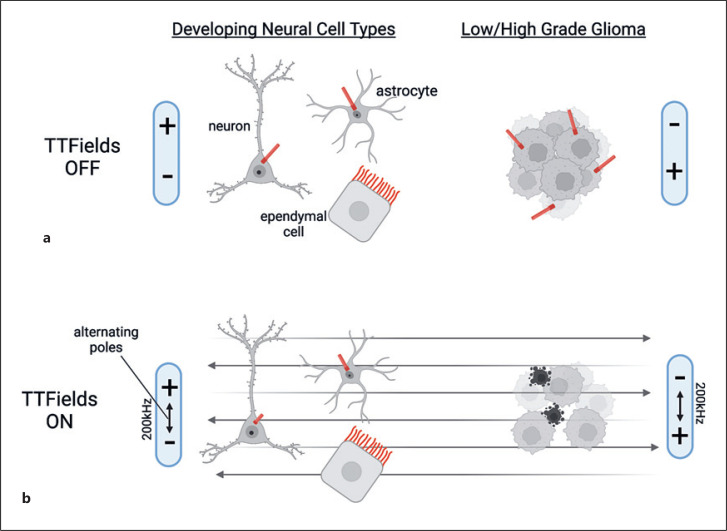
Impact of TTFields on cilia of developing neural cells versus glioma cells. **a** When TTFields are off, neurons, astrocytes, and ependymal cells and subsets of glioma cells bear primary or motile cilia (red). **b** When TTFields are turned on, in culture, neurons, astrocytes, and ependymal cells appear to hold onto their cilia during this process while glioma cilia are lost and cells begin to die. During TTFields, the positive and negative poles constantly flip to generate the alternating electric fields at 200 kHz across the cells. Image created using Biorender.com.

Not surprisingly, the cellular changes induced by TTFields are diverse, and to date there is no one attributable mechanism of action. For example, TTFields can alter microtubules during mitosis, increase DNA damage and replication stress, elicit autophagy and immunogenic cell death, reduce cell migration, and induce changes in cell permeability (for review, see [54]). Given the microtubule-based nature of cilia, our group was the first to examine whether primary cilia were sensitive to the effects of TTFields, in both cells cultured from primary mouse cortex and in low- or high-grade patient-derived glioma cells (Fig. 1). Strikingly, most ciliated glioma cells appear to lose or disassemble their cilium within 24 h of TTFields exposure [55]. If the treatment is halted, the cilia return to the abundance observed pre-TTFields. Surprisingly, neurons, astrocytes, multi-ciliated ependymal cells, and even proliferating cells (Ki67^+^) in mouse cortical cultures treated with TTFields were less affected (Fig. 1) [55]. After 24 h of TTFields, neuronal primary cilia frequency was unchanged, though the cilia lengths retracted some [55]. Even longer durations of TTFields did not have the same ciliary ablation effect on normal cell types [55]. The reasons for these differences are not clear but perhaps speak to some protective role of the cilium that would be activated by TTFields-induced stress, a cytoprotective mechanism that is lost in glioma cells.

**Fig. 2:**
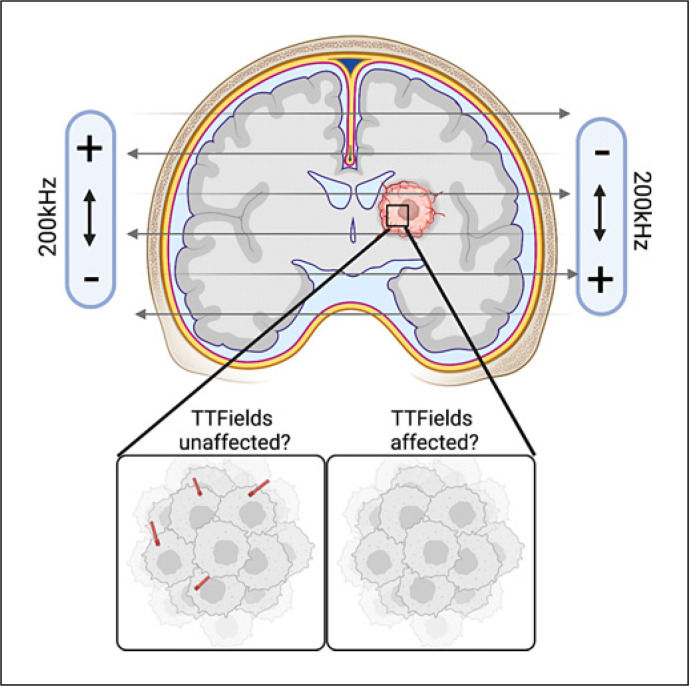
Unknown effects of TTFields on tumor cells bearing primary cilia deep in the human brain tumor microenvironment. Image created using Biorender.com.

Notably, treating freshly dissected patient tumors ex vivo overnight with TTFields also resulted in primary cilia loss in the tumor microenvironment [55]. It will be important to confirm if the loss of glioma cilia and preservation of ciliated normal cell types persists in vivo in rodent intracranial models of TTFields. However, the question remains if TTFields are powerful enough to have the same influence on tumor ciliogenesis embedded deep in the tissue (Fig. 2). In humans, the only way to assess this would be to perform systematic comparative analyses between initial, untreated biopsies and recurrent biopsies post-TTFields. Establishing a clinically relevant TTFields treatment of intracranial rodent tumor model will be paramount for a better understanding of the biological effects of this new therapeutic modality in the context of cilia modulation.

Supporting the notion that primary cilia are required for tumor growth after TTFields (or perhaps when the system is “off”), we observed return of ciliated populations post-TTFields, whether single or repeated exposures of TTFields. Thus, TTFields do not completely eliminate ciliated GBM cells. Whether ciliated tumors are more receptive to TTFields than tumors lacking cilia should be further tested in animal models.

## Primary Cilia on Glioma Cells Are Stimulated by TMZ and Promote Resistance to the Chemotherapy, a Process That Can Be Interfered with Using TTFields

**Fig. 3:**
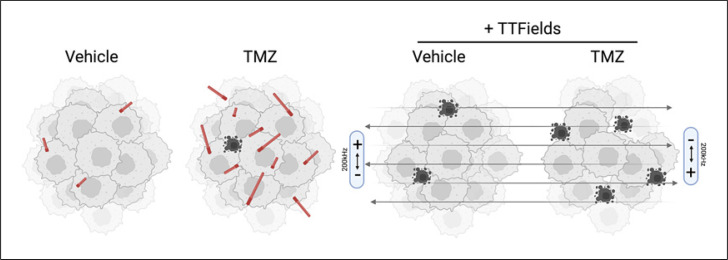
TMZ stimulates glioma ciliogenesis which is blocked by TTFields. Image created using Biorender.com.

Interestingly, multiple groups have found that inhibiting ciliogenesis on glioma cells sensitizes GBM cells to the effects of TMZ [42, 43, 55, 56]. Inhibiting expression of KIF3a, PCM1, ARL13B, or IFT88, all proteins required for ciliogenesis, can sensitize patient-derived tumor cells to TMZ in vitro and in vivo (e.g., [42]). Thus, the primary cilia on glioma cells are not only promoting growth of gliomas but also mediating signaling pathways that promote resistance to current chemotherapy. The molecular mechanisms exploited or triggered by the cilium are likely complex, including induction of de novo purine biosynthesis and/or autophagy processes [42, 43]. Whatever the mechanism, we and others have found that TMZ stimulates ciliogenesis, increasing both cilia length and frequency of ciliated GBM cells (Fig. 3) [43, 55]. The increase in ciliogenesis is in part due to epigenetic induction of *ARL13B* gene expression [42]. Thus, TMZ, while harmful and toxic to dividing cells, also appears counterproductive in the sense it stimulates tumor cells to become ciliated and supporting a more chemoresistant phenotype. This may also extend to stress induced by gamma irradiation which was recently shown to promote ciliogenesis in human GBM cells [43].

**Fig. 4:**
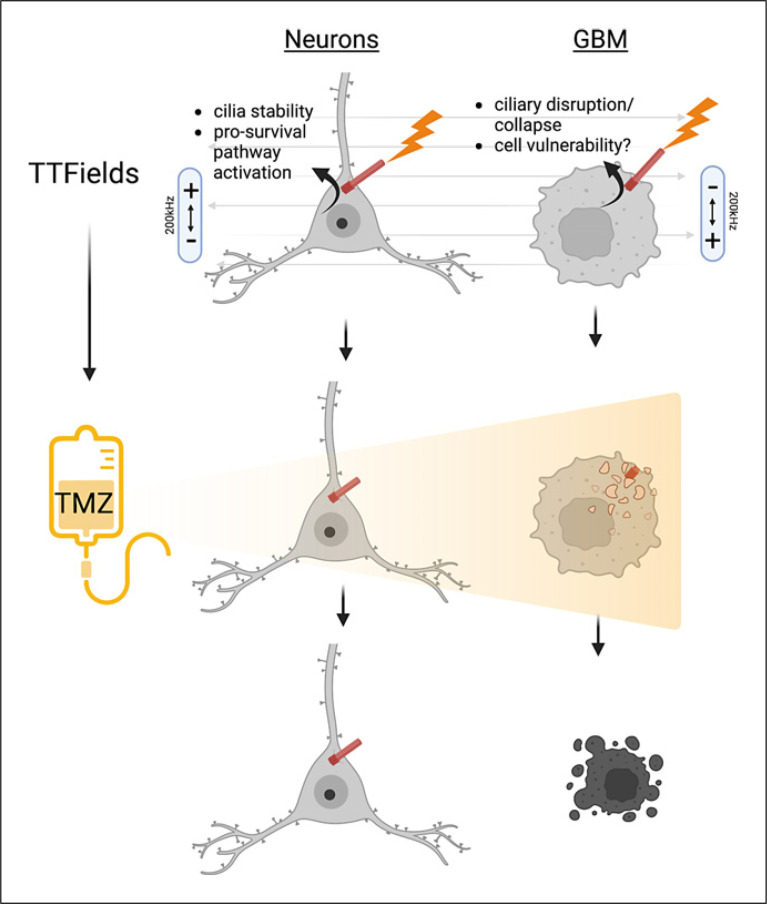
TTFields stimulation of neuronal versus glioma primary cilia: consequences and effects on TMZ sensitivity. TTFields may trigger different pathways in normal neural cell types (e.g., neurons) that are neuroprotective. Alternatively, GBM cells have altered underlying primary cilia that in the presence of TTFields, results in destabilization and collapse leading to TMZ sensitivity and ultimately cell death. The nature of the pathways induced at or involving cilia remains unknown. Image created using Biorender.com.

The induction of GBM ciliogenesis by TMZ raises an interesting question with respect to TTFields since the treatments have opposing influences on ciliogenesis and are co-administered to patients. Are primary cilia stimulated or inhibited when the treatments are applied concomitantly? In vitro, TTFields override the pro-ciliogenic effects of TMZ (Fig. 3) [55]. That is, the increase in GBM ciliogenesis does not occur while TTFields are being applied. We also found the co-application of both treatments led to inhibited expansion of cells after the treatments. However, the benefit of combined effects may depend on the relative timing of TMZ with respect to TTFields. For example, we found that adding TMZ after TTFields to a low-grade glioma cell line suppressed subsequent expansion of cells. However, simultaneous treatment of TTFields and TMZ did not suppress subsequent glioma cell expansion but rather enhanced it. The reason for this difference is unclear. Nevertheless, TTFields suppression of ciliogenesis sensitizes more cells to TMZ (Fig. 3), 4. Perhaps in neurons or other normal neural cell types, exposure to TTFields triggers different responses from the cilium, one that is activated and leads to prosurvival cues in neurons/other normal cell types, but in many GBM cells this pathway is lost, defective, or deactivated leading to ciliary disassembly and cell vulnerability (Fig. 4). Thus, there could be key signaling differences based on the nature of the TTFields stress that sets the stage for survival outcomes.

## Conclusion

Primary cilia appear to be at the crossroads regulating the effects of TTFields and TMZ treatments, at least from the results of in vitro and ex vivo studies. One treatment stimulates while the other ablates. However, a question remains if cilia are directly involved in the stress response or indirect readouts of other downstream changes induced by each therapy. The mechanisms need further exploration, validation, and determination of whether primary cilia are useful biomarkers for patient stratification and treatment efficacy. The data discussed in this review suggest efforts to suppress primary cilia may enhance therapeutic efficacy of TTFields and TMZ. However, this will need to be further tested and validated in more cell lines and animal models using agents that can pass the blood-brain barrier and break down GBM cilia.

Normal neural cell types appear spared from TTFields. Does this reflect a difference of plasma membrane permeability reported in normal cell types compared to GBM cells [57]? Or are TTFields/TMZ-related stress response pathways via the primary cilium different between normal and GBM cells? Future studies will again need in vivo models of intracranial TTFields application to determine whether in vitro findings accurately predict or model how the brain tumor microenvironment responds to these therapies. During such studies, available mouse models (e.g., where we can conditionally manipulate cilia in various neural cell types) may be able to shed light on whether TTFields and/or TMZ trigger different stress responses via cilia leading to survival and preservation of the brain circuitry for normal cell types while making GBM cells more vulnerable to combination therapies.

## Acknowledgments

The authors would like to thank Drs. Y. Porat, A. Haber, C. Higgins, and M. Giladi at Novocure Inc. for their review and comments on the manuscript.

## Conflict of Interest Statement

The work is in part supported by Novocure Inc. The corresponding author is a paid consultant for Novocure Inc.

## Funding Sources

M.R.S. is supported by a 2022 American Association for Cancer Research (AACR)-Novocure Tumor-Treating Fields Research (Grant No. #22-60-62-SARK). L.P.D. is supported by the NIH (R21NS116578 and 1R01NS121075) and the Florida Department of Health (22L06).

## Author Contributions

L.P.D. and M.R.S. wrote, edited, and approved the submitted version of this manuscript.
